# Multimorbidity and Its Relationship With Long-Term Outcomes After Critical Care Discharge: A Prospective Cohort Study

**DOI:** 10.1016/j.chest.2021.05.069

**Published:** 2021-06-18

**Authors:** Joanne McPeake, Tara Quasim, Philip Henderson, Alastair H. Leyland, Nazir I. Lone, Matthew Walters, Theodore J. Iwashyna, Martin Shaw

**Affiliations:** a Intensive Care Unit, Glasgow Royal Infirmary, Glasgow, Scotland; b MRC/CSO Social and Public Health Sciences Unit, University of Glasgow, Glasgow, Scotland; c School of Medicine, Dentistry and Nursing, University of Glasgow, Glasgow, Scotland; d Usher Institute, University of Edinburgh, Edinburgh, Scotland; e NHS Lothian, Edinburgh, Scotland; f Centre for Clinical Management Research, VA Ann Arbor Health System, Ann Arbor, MI; g Department of Internal Medicine, Division of Pulmonary and Critical Care, University of Michigan, Ann Arbor, MI; h Department of Clinical Physics and Bioengineering, NHS Greater Glasgow and Clyde, Glasgow, Scotland

**Keywords:** critical care, emotional, long-term mortality, readmission, HR, hazard ratio, IQR, interquartile range, IRR, incident rate ratio

## Abstract

**Background:**

Survivors of critical illness have poor long-term outcomes with subsequent increases in health care utilization. Less is known about the interplay between multimorbidity and long-term outcomes.

**Research Question:**

How do baseline patient demographics impact mortality and health care utilization in the year after discharge from critical care?

**Study Design and Methods:**

Using data from a prospectively collected cohort, we used propensity score matching to assess differences in outcomes between patients with a critical care encounter and patients admitted to the hospital without critical care. Long-term mortality was examined via nationally linked data as was hospital resource use in the year after hospital discharge. The cause of death was also examined.

**Results:**

This analysis included 3,112 participants. There was no difference in long-term mortality between the critical care and hospital cohorts (adjusted hazard ratio, 1.09; 95% CI, 0.90-1.32; *P* = .39). Prehospitalization emotional health issues (eg, clinical diagnosis of depression) were associated with increased long-term mortality (hazard ratio, 1.49; 95% CI, 1.14-1.96; *P* < .004). Health care utilization was different between the two cohorts in the year after discharge with the critical care cohort experiencing a 29% increased risk of hospital readmission (OR, 1.29; 95% CI, 1.11-1.50; *P* = .001).

**Interpretation:**

This national cohort study has demonstrated increased resource use for critical care survivors in the year after discharge but fails to replicate past findings of increased longer-term mortality. Multimorbidity, lifestyle factors, and socioeconomic status appear to influence long-term outcomes and should be the focus of future research.

## Graphical Abstract

**Figure:**
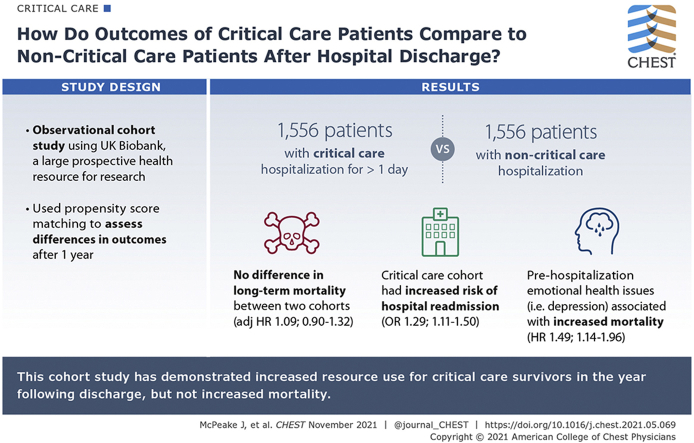

FOR EDITORIAL COMMENT, SEE PAGE 1587

The number of admissions to critical care internationally continues to steadily increase every year.^1^^,^^2^ Evidence demonstrates that discharge from critical care is often the start of a challenging recovery trajectory for both patients and caregivers, including physical, social, cognitive, and emotional problems in the years after discharge.^3^, ^4^, ^5^

Excess mortality and increased health care utilization have also been reported in the post-hospital period.^6^^,^^7^ Patients who have been diagnosed with sepsis and those with worse pre-critical care physical health are particularly at risk of poorer outcomes; however, such excess long-term mortality was not present for hypoxic respiratory failure.^8^, ^9^, ^10^ However, beyond these average population effects, there is limited information about the variation in patients based on their preexisting social circumstances and mental health problems, and the impact that these may have on longer-term outcomes.

Using data from the UK Biobank, we sought to advance the evidence by answering the following three questions: (1) do critical care patients have a different mortality rate or readmission risk use in comparison with hospitalized patients who do not need care in a critical care environment?; (2) what are the causes of death in the post-critical care period?; and (3) what is the interplay between mental and social health issues and health care utilization after admission to critical care?

## Methods

### Data and Patients

We reported an observational cohort study as per the STrengthening the Reporting of OBservational studies in Epidemiology guidelines.^11^

Data were obtained from the UK Biobank, a large prospective health resource for research which aims to improves the prevention, diagnosis, and treatment of a range of illnesses. Between 2006 and 2010, the UK Biobank recruited > 500,000 participants from the UK population.^12^ Those participants enrolled and attended assessment centers across the United Kingdom, where they completed a wide range of assessments alongside in-depth objective physical measurement.

The UK Biobank study was approved by the North West Multicentre Ethics Research Committee; participants provided written informed consent and agreed to have their health followed longitudinally, via linkage to routine clinical data (including health care resource use and diagnostic data). This study is part of UK Biobank project 57617 (NHS National Research Ethics Service No. 11/NW/0382). Data for this analysis were extracted from the UK Biobank server on October 22, 2020 (censor date). All patients who have withdrawn consent from the UK Biobank were removed from this analysis. Data in the UK Biobank are linked to routinely held NHS data annually.

### Study Cohorts

**Figure 1:**
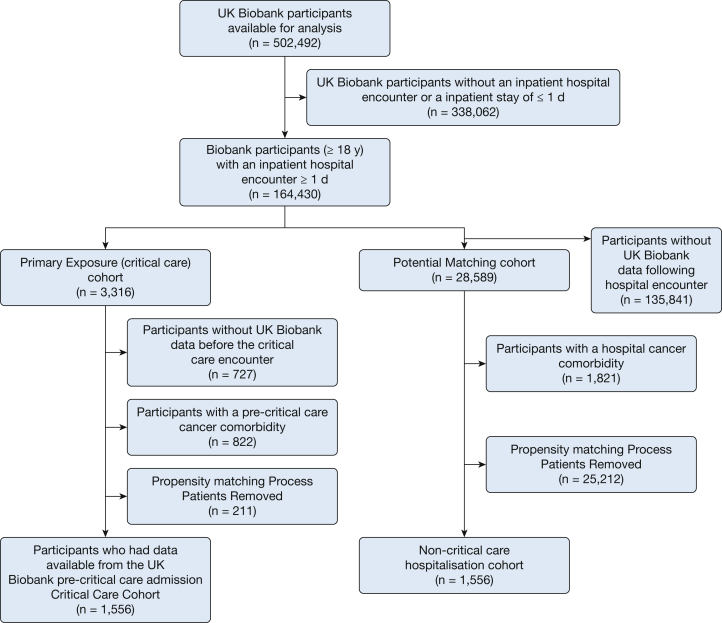
Flowchart of UK Biobank participants included in this study.

Two study cohorts were created from the UK Biobank (Fig 1). The primary cohort in this analysis included participants with a critical care admission who had UK Biobank data available before critical care admission (critical care cohort). The critical care cohort was defined by consultant specialty within the UK Biobank dataset (e-Table 1). We considered the first critical care and first hospital admission for the comparison cohorts to accurately reflect baseline status. We used data from the immediate, preceding UK Biobank assessment center visit to the admission. The second cohort was a group of hospitalized patients with similar baseline characteristics not admitted to critical care (hospital cohort). Only participants ≥ 18 years of age at the time of their critical care/hospital admission, who had been admitted to hospital for ≥ 1 day, were included.

Participants who had a previous cancer diagnosis (solid tumor without metastasis, malignancy [including lymphoma and leukemia except malignant neoplasm of skin], lymphoma, metastatic cancer or metastatic solid tumor) were excluded from both cohorts because patients with cancer are known to have a different recovery trajectory than patients without cancer after critical care discharge.^13^ This exclusion included a diagnosis of cancer before or during the index admission. Participants in this analysis were admitted to critical care between 2006 and 2017.

### Demographics

Area-level socioeconomic deprivation was assessed by the Townsend Deprivation Index, corresponding to the output area in which the respondent’s home postcode was recorded.^14^ Comorbidities were classified using the Elixhauser Comorbidity Index and the Charlson Comorbidity Index.^15^^,^^16^ A clinical diagnosis of depression was included as a comorbidity within the Elixhauser Comorbidity Index. Comorbidities were included in this analysis if they had been diagnosed before or at the hospital/critical care admission. Furthermore, only comorbidities which had been diagnosed during an acute hospital encounter were included, with the aim that this study characterized the clinically important comorbidities. Comorbidities are integrated into the UK Biobank via routinely collected national hospital data. The comorbidities used are listed in e-Table 2. Ethnicity was also recorded. Because educational attainment has been shown to be important during recovery from critical illness, we included it in our analysis.^17^

### Development of Cohort Matching Models

We chose to use propensity score matching (based on critical care admission) using a nearest neighbor methodology to achieve a cohort with optimal common support in the analytical sample.^18^ A caliper width of 0.25 of the SD of the logit of the propensity score was used for the development of matching to adequately capture an appropriate sample. Matching was undertaken at a 1:1 ratio using age (age at hospital/critical care admission), sex, admission type (emergency, elective, surgical [including first surgical procedure, if present], and medical), hospital length of stay, the Townsend Index, ethnicity, educational attainment of the participant, presence of multimorbidity (two or morbidities from e-Table 2), smoking status, and date between UK Biobank assessment visit and admission to critical care. When matching, we also considered data which were obtained from the UK Biobank assessment center for participants before the index critical care/hospital admission. Only data for the immediately preceding UK Biobank assessment center visit (in relation to critical care/hospital admission) were used. This included the following: features of anxiety (presence of nervous feelings), FVC (liters), employment status, and self-reported social isolation. Information on how these covariates were calculated has been previously published with full details available in e-Table 3.^19^ Differences between the two cohorts were evaluated using either the Pearson χ^2^ test or a Kruskal-Wallis test. Nonnormal variables were log-transformed before matching.

### Outcomes

The primary outcome was mortality during follow-up. Mortality information for both cohorts was obtained from the date of death which is available via the UK Biobank. The primary cause of death was obtained via linkage to national registries (also via UK Biobank), using the *International Classification of Diseases, Tenth Revision* code. These data are updated annually within the UK Biobank. Data on mortality were available from the UK Biobank until July 2020. We also measured rates of readmission to hospital in the year after critical care discharge.

### Statistical Analysis

We conducted all analyses with R (version 4.0.2; R Core Team). All missing covariates were imputed via a single imputation using predictive mean matching with the Multivariate Imputation by Chained Equations software package (Stef van Buuren). Each variable with missing values was regressed on all other analyzed variables. Data on missing values are presented in e-Table 4. Results of comparisons with *P* ≤ .05 were considered to represent statistically significant differences.

#### Long-term Mortality

We generated survival curves for the critical care and the hospital cohorts. Cox proportional hazards models, stratified by matched pairs, were used to estimate hazard ratios (HRs) for mortality in the critical care vs hospital control participants, adjusted for potential confounders. Potential confounders were identified from the literature and collinear variables removed.^20^^,^^21^ Cohort comparisons and the survival curves were both estimated using predictions from the Cox proportional hazard model. Variables with *P* < .10, determined using two-tailed, paired testing on univariate analysis, were entered in the final adjusted models. The results were expressed as HRs with a corresponding 95% CI and interquartile range (IQR). Because of the large number of participants with preexisting comorbidities included, we undertook a sensitivity analysis which explored whether the number of comorbidities preadmission to critical care impacted longer-term mortality. We defined multimorbidity has having two or more documented comorbidities.

#### Cause of Death

Reason for death was displayed as counts. We examined the primary cause of death from the cohorts using death records and examined the frequency of cause.

#### Readmission and Health Care Utilization

We estimated incident rate ratios (IRRs) using negative binomial regression to model the number of hospital admissions in the year post-critical care discharge. Potential confounders were included in the multivariable model, similar to the approach undertaken for the mortality analysis. Logistic regression models were used to determine the risk of being readmitted to hospital within 1 year of discharge. The results were expressed in terms of the OR with a corresponding 95% CI. A readmission was defined as a hospital stay of > 24 h.

## Results

### Cohort Characteristics

From the 502,492 UK Biobank participants, 1,767 participants had a Biobank assessment visit before a critical care admission. We were able to successfully match this critical care group with 1,556 participants (88.1%) with a non-critical care hospitalization (Fig 1). The standardized mean differences between the matched and unmatched cohorts are presented in e-Figure 1.

In the critical care cohort, the median age was 66 years (IQR, 60-71 years), 899 (57.8%) were men, 1,472 (94.6%) were white, and 943 (60.6%) had two or more comorbidities. The median time from the UK Biobank assessment to admission to critical care was 1,718 days (IQR, 985-2,386 days). The median critical care length of stay was 2 days (IQR, 1-4 days). In the hospital cohort, the median age was 66 years (IQR, 60-71 years), 897 (57%) were men, and 964 (62%) had two or more comorbidities. The median time between UK Biobank assessment and admission was 1,755 days (IQR, 943-2,397 days). Between the two matched cohorts, the only statistically significant difference was total hospital length of stay (9 [critical care] vs 8 [hospital] days, *P* = .04) (Table 1).

**Table 1:** Baseline Demographics of the Critical Care Cohort Compared With the Hospital Control Cohort

Demographic	Hospital Cohort (n = 1,556)	Critical Care Cohort (n = 1,556)	*P* Value
Age, y	66 (60 to 70)	66 (60 to 71)	.83
Sex, male	887 (57)	899 (57.8)	.66
Ethnicity			
White	1,470 (94.5)	1,472 (94.6)	.89
Mixed	14 (0.9)	11 (0.7)	
Other South Asian	8 (0.5)	6 (0.4)	
Black	24 (1.5)	21 (1.3)	
Chinese	6 (0.4)	a (0.2)	
Indian	19 (1.2)	24 (1.5)	
Pakistani	7 (0.5)	8 (0.5)	
Other	8 (0.5)	11 (0.7)	
Comorbidities (two or more)	964 (62)	943 (60.6)	.44
Highest education attainment			.98
College/university degree	377 (24.2)	374 (24)	
Other professional qualification	245 (15.8)	241 (15.5)	
A levels/AS levels	101 (6.5)	96 (6.2)	
NVQ/HND/HNC	187 (12)	186 (12)	
O levels/GCSEs/CSEs	249 (16)	265 (17)	
None of the above	397 (25.5)	394 (25.3)	
Townsend Index	−1.48 (−3.31 to 2.02)	−1.50 (−3.37 to 1.89)	.48
Smoking status			.97
Current	287 (18.4)	289 (18.6)	
Previous	605 (38.9)	610 (39.2)	
Never	664 (42.7)	657 (42.2)	
Days between assessment and critical care/hospital admission	1,755 (943 to 2,397)	1,718 (985 to 2,386)	.87
Admission type, surgical	1,360 (87.4)	1,379 (88.6)	.29
Admission type, emergency	1,159 (74.5)	1,113 (71.5)	.06
Hospital length of stay, d	8 (4-22)	9 (4-21)	.04
Social isolation	13 (0.8)	8 (0.5)	.27
Nervousness	369 (23.7)	393 (25.3)	.32
Employment status			.92
Not defined	18 (1.2)	20 (1.3)	
Unable to work	183 (11.7)	191 (12.3)	
Vocational	11 (0.7)	13 (0.8)	
Purposeful	1,344 (86.4)	1,332 (85.6)	
FVC, L	3.31 (2.68 to 3.98)	3.33 (2.68 to 4.06)	.64

Values are No. (%), median (interquartile range), or as otherwise indicated. CSE = certificate of secondary education; HND = higher national diploma; HNC = higher national certificate; GCSE = general certificate of secondary education; Not defined = not answered or did not fit into any other category; NVQ = national vocational qualification; Purposeful = employed or retired; Unable to work = sickness/disability or unemployed; Vocational = doing unpaid or voluntary work or student.

a Denotes a value of less than five.

### Long-term Mortality

Minimum post-hospital discharge follow-up time was 1,218 days and the maximum was 4,869 days (median, 2,364 days; IQR, 1,774-3,063 days). Hospital mortality in the critical care group was 14.8% vs 3.3% in the hospital cohort. Mortality for the critical care cohort at 30 days post-hospital discharge was 15.9% vs 4.5% in the hospital cohort. At 1 year this was 19.7% vs 8.5%, and at 3 years this was 25.8% vs 13.1%, respectively (Table 2).

**Table 2:** Cumulative Mortality in the Critical Care and Hospital Cohorts

Time Frame	Hospital Cohort Survival, No. (%)	Mortality (%)	Cumulative Hazard (95% CI)	Critical Care Cohort Survival (%)	Mortality (%)	Cumulative Hazard (95% CI)
Admission to hospital	1,556 (100)	…	…	1,556 (100)	…	…
Critical care discharge	…	…	…	1,469 (94.4)	5.6	…
Discharge	1,505 (96.7)	3.3	…	1,325 (85.2)	14.8	…
30 d postdischarge	1,486 (95.5)	4.5	0.07 (0.00-0.15)	1,309 (84.1)	15.9	0.08 (0.00-0.17)
60 d postdischarge	1,474 (94.7)	5.3	0.48 (0.23-0.72)	1,301 (83.6)	16.4	0.52 (0.25-0.79)
1 y postdischarge	1,423 (91.5)	8.5	3 (2.0-4.0)	1,249 (80.3)	19.7	3.30 (2.17-4.43)
3 y postdischarge	1,350 (86.8)	13.1	7.5 (5.5-9.9)	1,154 (74.2)	25.8	8.32 (5.72-10.90)
5 y postdischarge	1,284 (82.5)	17.5	12.0 (8.6-15.3)	1,095 (70.4)	29.6	13 (9-17)
10 y postdischarge	1,228 (78.9)	21.1	24.0 (17.2-30.8)	1,026 (65.9)	34.1	26.1 (18.0-34.2)
> 11 y postdischarge	1,225 (78.7)	21.3	…	1,024 (65.8)	34.2	…

**Figure 2:**
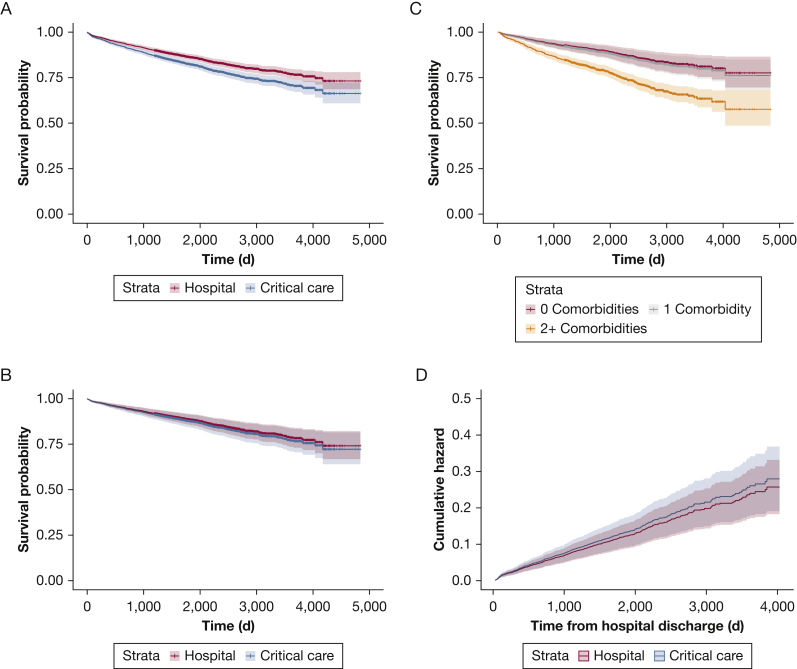
A-D, Survival plots describing the long-term outcomes of the hospital and critical care cohorts. A, Unadjusted survival model (hospital vs critical care cohort). B, Adjusted survival model (hospital vs critical care cohort). C, Adjusted survival model (multimorbidity. D, Cumulative mortality hazard following discharge (hospital vs critical care).

After adjustment, there was no significant difference in longer-term (post-hospital) mortality between the critical care and hospital cohort (HR, 1.09; 95% CI, 0.90-1.32; *P* = .39) (Fig 2) across the follow-up period. Among the participant characteristics assessed, emergency admissions, increased age, longer index hospital length of stay, and male sex increased long-term mortality. Comorbidities (eg, renal disease, liver disease) were associated with increased mortality, alongside a diagnosis of clinical depression (adjusted HR, 1.49; 95% CI, 1.14-1.96; *P* < .004). Current and previous smoking status also had a significant impact on survival (adjusted current smoking status: HR, 1.82; 95% CI, 1.43-2.32; *P* < .001) (Table 3). Socioeconomic deprivation, measured through the Townsend Deprivation Index, increased long-term mortality (HR, 1.31; 95% CI, 1.13-1.53; *P* < .001), as did having less people living in the participants household (HR, 0.92; 95% CI, 0.85-0.99; *P* = .046).

**Table 3:** Adjusted HR and Unadjusted HR Survival Analysis

Variable		Unadjusted HR (95% CI)	Unadjusted *P* Value	Adjusted HR (95% CI)	Adjusted *P* Value
Age		1.06 (1.04-1.07)	< .001	1.06 (1.04-1.07)	**< .001**
Critical care		1.32 (1.11- 1.56)	.001	1.09 (0.90-1.32)	.39
Sex	Male	1.31 (1.10- 1.56)	.002	1.35 (1.09- 1.68)	**.007**
GP for anxiety/depression		1.16 (0.98- 1.38)	.09	0.91 (0.74-1.1)	.36
Nervous feelings		1.22 (1.01- 1.47)	.04	1.19 (0.96- 1.49)	.12
Tense feelings		1.23 (1.00- 1.51)	.05	0.99 (0.78- 1.26)	.95
Loneliness		1.02 (0.84- 1.23)	.87	…	…
Overall health	Fair	0.73 (0.57- 0.94)	.01	0.87 (0.66- 1.15)	.33
	Good	0.56 (0.44- 0.71)	< .001	0.77 (0.57- 1.04)	.09
	Excellent	0.30 (0.20- 0.46)	< .001	0.49 (0.30- 0.78)	**.003**
Employment status	Unable to work	1.49 (0.60- 3.69)	.39	…	…
	Vocational	1.35 (0.36- 5.04)	.65	…	…
	Purposeful	1.38 (0.57- 3.33)	.47	…	…
Government allowances	Blue badge	1.76 (1.04-3.00)	.04	1.24 (0.71- 2.16)	.44
	Allowances	1.73 (1.40-2.15)	< .001	0.96 (0.74-1.25)	.79
No. in household		0.84 (0.78-0.91)	< .001	0.92 (0.85- 0.998)	**.046**
Hand grip strength		0.99 (0.99- 1.00)	.18	…	…
FVC		0.82 (0.75- 0.89)	< .001	0.86 (0.77- 0.96)	**.02**
Summed minutes activity		1 (1-1)	.01	1 (1-1)	.55
Length of stay (full)		1 (1-1)	.07	1. (1-1)	**.003**
Emergency admission		1.52 (1.25- 1.86)	< .001	1.34 (1.09- 1.65)	**.006**
Surgical admission		1.12 (0.86-1.45)	.42	…	…
Townsend Deprivation Index		1.06 (1.03-1.08)	< .001	1.04 (1.01- 1.07)	**.005**
Ethnic background	Mixed	0.40 (0.1- 1.59)	.19	…	…
	South Asian	1.12 (0.36- 3.50)	.84	…	…
	Black	1.13 (0.59-2.18)	.72	…	…
	Chinese	0.54 (0.08- 3.80)	.53	…	…
	Other	0.34 (0.05- 2.42)	.28	…	…
	Indian	1.24 (0.64- 2.39)	.53	…	…
	Pakistani	1.10 (0.35- 3.42)	.87	…	…
Qualifications	CSEs	0.47 (0.27- 0.81)	.007	0.75 (0.43- 1.30)	.30
	O levels/GCSEs	0.64 (0.48-0.86)	.003	0.93 (0.69- 1.26)	.64
	NVQ/ HND or HNC	0.61 (0.45-0.82)	.001	0.83 (0.61- 1.13)	.24
	A levels/AS levels	0.61 (0.42-0.9)	.012	0.94 (0.64-1.39)	.76
	Other professional	0.57 (0.43-0.75)	< .001	0.78 (0.59- 1.04)	.09
	College/university	0.72 (0.57-0.90)	.004	1.07 (0.84-1.36)	.58
Smoking status	Current	1.89 (1.52- 2.36)	< .001	1.82 (1.43- 2.32)	**< .001**
	Previous	1.41 (1.16- 1.71)	< .001	1.24 (1.01-1.52)	**.037**
Comorbidities					
	Hypothyroidism	0.85 (0.60- 1.19)	.34	…	…
	Cardiac arrhythmias	1.37 (1.05-1.79)	.02	1.07 (0.81- 1.42)	.62
	Deficiency anemia	1.52 (1.03- 2.26)	.04	0.97 (0.64-1.48)	.90
	Chronic pulmonary disease	1.45 (1.18- 1.77)	< .001	0.99 (0.79- 1.23)	.90
	Diabetes (no complications)	1.36 (1.12-1.66)	.002	1.05 (0.84- 1.31)	.70
	Myocardial infarction	1.17 (0.90- 1.51)	.24	…	…
	Neurologic disorders	2.45 (1.9- 3.15)	< .001	2.12 (1.6-2.82)	**< .001**
	Pulmonary circulation	1.66 (1.10- 2.5)	.02	1.42 (0.93- 2.19)	.11
	Cerebrovascular disease	1.28 (1.0- 1.65)	.05	0.93 (0.71- 1.21)	.59
	Obesity	1.05 (0.79- 1.4)	.75	…	…
	Alcohol abuse	1.25 (0.95- 1.63)	.11	…	…
	Congestive heart failure	1.83 (1.42- 2.37)	< .001	1.25 (0.94- 1.66)	.13
	Fluid/electrolyte disorders	1.66 (1.28- 2.14)	< .001	1.02 (0.77- 1.35)	.90
	Hypertension complicated	1.80 (1.21- 2.70)	.004	0.71 (0.40- 1.24)	.23
	Renal disease	1.81 (1.35- 2.41)	< .001	1.56 (1.05- 2.32)	**.03**
	Valvular disease	1.32 (0.99- 1.77)	.06	1.02 (0.74- 1.40)	.91
	Peripheral vascular disorder	1.37 (1.04- 1.81)	.02	0.89 (0.66- 1.20)	.44
	Weight loss	2.27 (1.51- 3.42)	< .001	1.59 (1.04- 2.44)	**.03**
	Hemiplegia or paraplegia	1.00 (0.56- 1.77)	.995	…	…
	Dementia	4.97 (3.32- 7.44)	< .001	3.05 (1.98- 4.71)	**< .001**
	Peptic ulcer disease	1.55 (1.02- 2.35)	.04	1.09 (0.69- 1.73)	.70
	Diabetes (complicated)	3.15 (2.15- 4.61)	< .001	1.84 (1.16- 2.91)	**.01**
	Rheumatic disease	1.35 (0.93- 1.97)	.11	…	…
	Depression	1.70 (1.34- 2.16)	< .001	1.47 (1.13- 1.93)	**.005**
	Mild liver disease	2.45 (1.82- 3.29)	< .001	1.93 (1.37- 2.73)	**< .001**
	Moderate/ severe liver disease	3.39 (2.03- 5.66)	< .001	1.74 (0.91- 3.32)	.10
	Coagulopathy	1.64 (0.94- 2.84)	.08	1.07 (0.57-2.01)	.83
	Blood loss anemia	0.55 (0.08- 3.93)	.55	…	…
	Psychoses	1.41 (0.77- 2.55)	.26	…	…
	Drug abuse	1.49 (0.48- 4.64)	.49	…	…

Significant multivariable *P* values shown in bold. CSE = certificate of secondary education; GCSE = general certificate of secondary education; GP = general practitioner; HNC = higher national certificate; HND = higher national diploma; HR = hazard ratio; NVQ = national vocational qualification.

We also considered the in-hospital variables available as mediators in a sensitivity analysis. We removed all hospital factors in the adjusted survival model (length of stay, nature of admission, and whether the admission was an emergency). This model also demonstrated that there was no significant difference in longer-term mortality between the critical care and hospital cohort (HR, 1.04; 95% CI, 0.86-1.26; *P* = .68) (e-Table 5).

In relation to multimorbidity, mortality was more than double in those participants with two or more documented comorbidities compared with those without comorbidities (HR, 2.17; 95% CI, 1.51-3.13; *P <* .001). There was no difference in mortality between the critical care participants with one comorbidity in comparison with those with no comorbidities (HR, 1.07; 95% CI, 0.68-1.68; *P =* .77) (Fig 2). In a further sensitivity analysis, we evaluated the impact of missing data on the final adjusted survival model. Removing all variables with missing data of > 10% did not change the reported difference in mortality between the two cohorts (e-Table 6).

### Cause of Death

**Figure 3:**
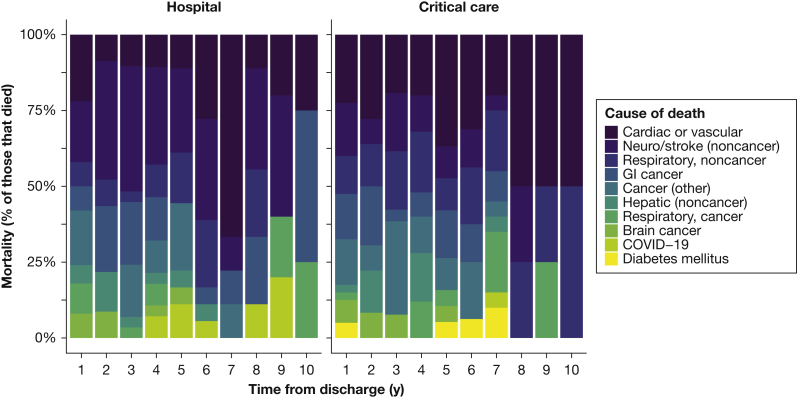
Breakdown of causes of death after critical care over time.

We examined the cause of death in both cohorts after 30 days post-hospital discharge. There were 261 deaths in the hospital cohort vs 285 deaths in the critical care cohort (e-Table 7). There were no clinical differences in cause of death across the two cohorts after 30 days discharge (Fig 3).

### Hospital Readmission and Health Care Utilization

In the year after critical care/hospital discharge, the number of patients with at least one readmission to hospital in the critical care cohort was significantly higher than the hospital cohort (50.8% vs 41.9%, respectively; *P* < .01). The median number of readmissions was also significantly higher in the critical care cohort (1; IQR, 0-2 vs 0; IQR, 0-1; *P* < .01) (Table 2).

The relative rate of hospital readmission was almost 30% higher in the critical care cohort than the hospital cohort (IRR, 1.27; 95% CI, 1.12-1.42; *P* < .001). Current smokers had 30% more readmissions (IRR, 1.30; 95% CI, 1.09-1.55; *P* = .004) than those who had never smoked. Participants with comorbidities (eg, liver disease, renal disease, cerebrovascular disease) also had more readmissions (e-Table 8).

After adjustment, the critical care cohort had a 29% increased risk of a hospital readmission than the hospital cohort in the year after discharge (OR, 1.29; 95% CI, 1.11-1.50; *P* = .001) (e-Table 9). The highest proportion of readmission took place in the first 120 days post-hospital discharge (e-Fig 2). However, the critical care cohort continued to have an increased volume of readmissions during the entire follow-up period.

## Discussion

This study reports the results of a prospective, nationally linked cohort study. Using data from the UK Biobank, we have demonstrated that the long-term mortality of critical care participants was no different from a propensity matched hospital cohort, after adjustment for baseline physical, social, and emotional health status. Consistent with previous research, readmissions in the year after hospital discharge were significantly higher in the critical care cohort.

Previous research has shown survivorship from sepsis and critical care is associated with higher long-term mortality.^6^^,^^8^ The deviation in this study may be caused by the use of socioeconomic and mental health demographics, which have not been used previously. Uniquely, we were also able to access data on household occupancy. The influence of social isolation and social relationships on the risk of death are comparable with well-established risk factors for mortality (eg, smoking, alcohol consumption) and may exceed the influence of other risk factors (eg, physical inactivity, obesity).^22^ The association between lower socioeconomic status and higher short- and long-term mortality after critical illness has also been described previously.^23^ This study reflects these important concepts: the social circumstances in which people live are important to recovery from critical care and should be prioritized both during and after the critical care encounter through appropriate rehabilitation and mental health services.

A strength of this study was the inclusion of depression as a clinically diagnosed comorbidity, rather than a self-reported variable. Using this definition, we have demonstrated that those patients with a history of clinical depression before hospital/critical care have worse long-term outcomes in comparison with those without this diagnosis. The bidirectional nature of physical and mental health is well established; patients with mental health problems are more likely to develop physical health problems. Similarly, mental health problems can become problematic in those with long-term physical morbidity.^24^ Furthermore, mental health issues become even more complex in multimorbidity and as socioeconomic deprivation worsens.^25^

Although the challenges associated with deprivation, multimorbidity, mental health issues, and isolation may not immediately seem amendable to critical care physician input, there may be changes to practice which could influence outcomes and provide long-term patient benefit. First, the information we provide during and after the critical care stay should be clear, accessible, and adapted for patients and caregivers of all backgrounds. Health literacy, defined as the cognitive and social skills which support confidence and ability in individuals to gain access to understand and use information in ways which promote and maintain good health, is known to be lower in people from disadvantaged and minority backgrounds.^26^ By empowering and providing patients and families with accessible and timely information about their health status during and after critical illness, we may see improvements in health-related behaviors and outcomes. Second, we must also ensure that we are not exacerbating preexisting mental health problems. For example, a multicenter study demonstrated that psychiatric drugs were commonly mismanaged during critical illness.^27^ Physicians must ensure that mental health and social comorbidities are given the same priority as physical comorbidities during the acute illness journey. Finally, research has demonstrated that a critical care admission may represent a teachable moment in those with alcohol misuse.^28^ Engaging addiction workers and other support teams as appropriate during the critical care admission may have long-lasting beneficial effects.

A further mechanism by which critical care physicians can potentially support multimorbidity and the effects of deprivation is through the provision of ICU aftercare, which crosses health and social care boundaries. New financial and social problems are common in the ICU recovery period; as such, including social workers and welfare advisors in ICU follow-up could support new and preexisting problems.^29^, ^30^, ^31^ The provision of peer support programs embedded in, or run-in tandem with critical care clinics, may also diminish the negative impact of loneliness and social isolation.^32^

UK Biobank participants admitted to critical care had a 29% increased risk of a hospital admission in the year after critical care discharge. Qualitative evidence has also shown the impact of patient-level issues (eg, multimorbidity, socioeconomic deprivation) alongside system-level issues (eg, poor discharge planning) on unplanned hospital readmissions.^33^ Research is required to fully understand how systems can support individuals to recover safely in the community and avoid any unnecessary readmissions to acute care.

The UK Biobank has provided an opportunity to examine the impact of multimorbidity on longer-term mortality and health care utilization. The strengths of this study are its use of unique precritical care markers, which were used to understand longer-term mortality and health care utilization. However, this study does have limitations. First, although we have information on admission type and consultant care, we have limited data about the severity of illness of participants and other critical care severity illness markers because these data were not available. Second, we examined comorbidities diagnosed before and at the index admission from routine data collection; we do not know the severity of these comorbidities or if they were active at the time of admission. Moreover, the median time between UK Biobank assessment and critical care admission was 1,718 days. The health of participants could have changed during this time frame. Also, the overall recruitment to the UK Biobank was only around 5.5%. The characteristics of this group may vary considerably from the wider population. Third, a large proportion of the patients included in this study, and indeed the UK Biobank as a whole, were surgical admissions; the outcomes noted in this paper may be different for medical critical care populations. Although > 70% of these patients were deemed to be emergency patients, caution must be taken when interpreting these results because they do not necessarily reflect all emergency admissions to critical care. Additionally, participants of the UK Biobank are on average healthier and are more likely to live in less socioeconomically deprived areas than the general UK population, which may limit the results.^34^

## Interpretation

After adjustment for prehospital physical, social, and emotional variables, long-term mortality in critical care patients does not appear to be any different in comparison with a hospital control comparator group. However, health care utilization was different between the two cohorts in the year after discharge, with the critical care cohort requiring a higher rate of rehospitalization. Multimorbidity, lifestyle factors, and socioeconomic status were significantly associated with both increased mortality and health utilization and should be the focus of future research.

**Take-home Points**

**Study Question:** Do critical care patients have a different mortality rate or readmission risk in comparison with hospitalized patients who do not need care in a critical care environment?

**Results:** Using data from > 3,000 patients, we have demonstrated that critical care patients are more likely to be readmitted to hospital in the year after discharge compared with a matched hospital control cohort. However, there was no difference in long-term mortality between the critical care and hospital cohorts.

**Interpretation:** This cohort study has demonstrated increased resource use for critical care survivors in the year after discharge, but not increased mortality.

## Supplementary Data

e-Online Data

Audio
