# Supplementary material for: Multimorbidity and Its Relationship With Long-Term Outcomes After Critical Care Discharge: A Prospective Cohort Study
Source: Chest. 2021 Jun 18;160(5):1681–92. doi: 10.1016/j.chest.2021.05.069 (PMC9199363; doi:10.1016/j.chest.2021.05.069)
Supplement: e-Online Data [file mmc1.pdf]

CHEST Online Supplement

Multimorbidity and Its Relationship With Long-Term Outcomes After Critical Care Discharge

A Prospective Cohort Study

Joanne McPeake, PhD; Tara Quasim, MD; Philip Henderson, MBcHB; Alastair H. Leyland, PhD; Nazir I. Lone, PhD; Matthew Walters, MD; Theodore J. Iwashyna, PhD; and Martin Shaw, PhD

CHEST 2021; 160(5):1681-1692

Online supplements are not copyedited prior to posting and the author(s) take full responsibility for the accuracy of all data.

© 2021 AMERICAN COLLEGE OF CHEST PHYSICIANS. Reproduction of this article is prohibited without written permission from the American College of Chest Physicians. See online for more details. **DOI:** 110.1016/j.chest.2021.05.069

e-Figure 1: Love plot of standardised mean difference in matching covariates

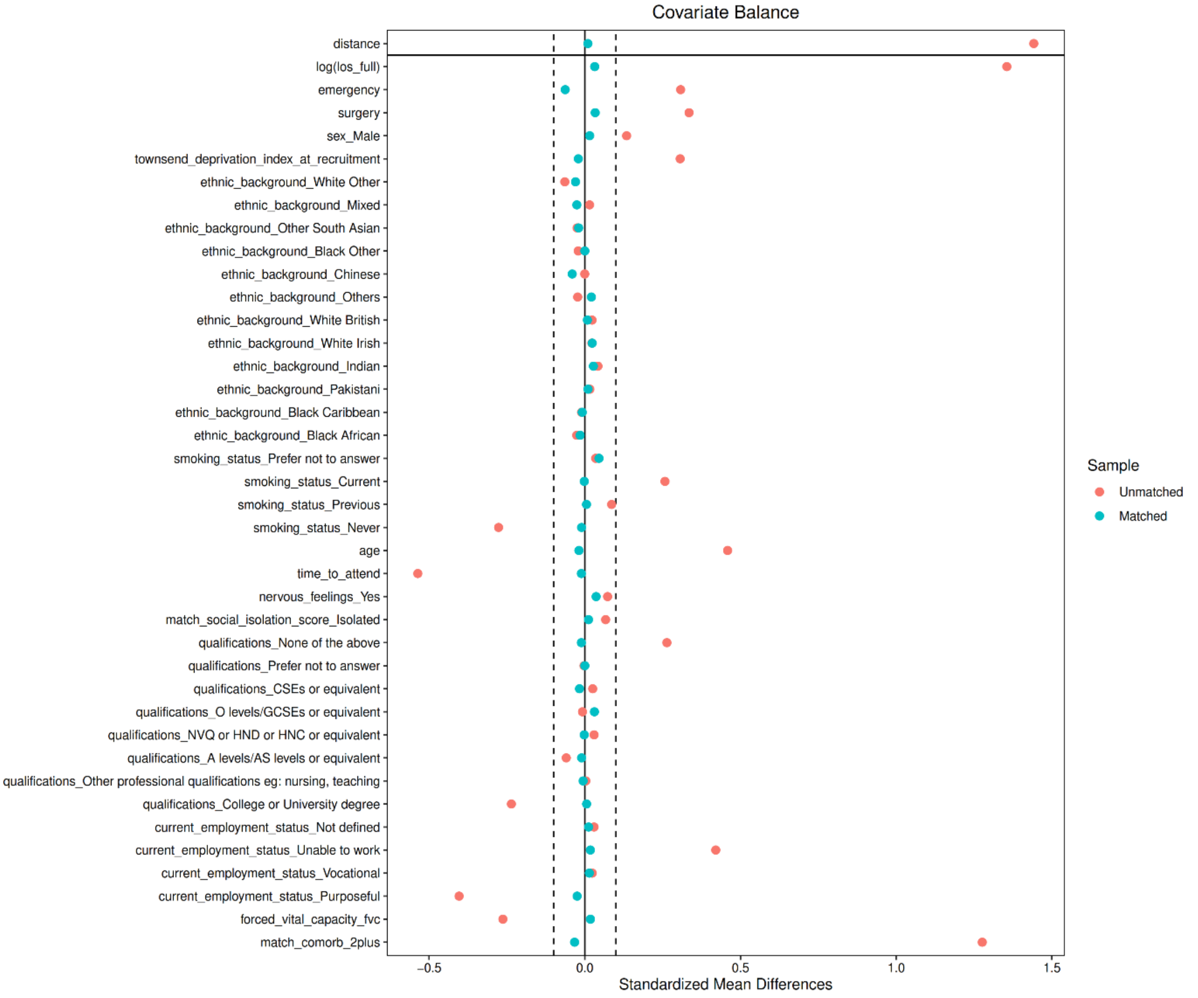

Online supplements are not copyedited prior to posting and the author(s) take full responsibility for the accuracy of all data.

CHEST® Online Supplement

e-Figure2: Count of hospital readmissions in the critical care vs hospital control group over the entire follow–up period.

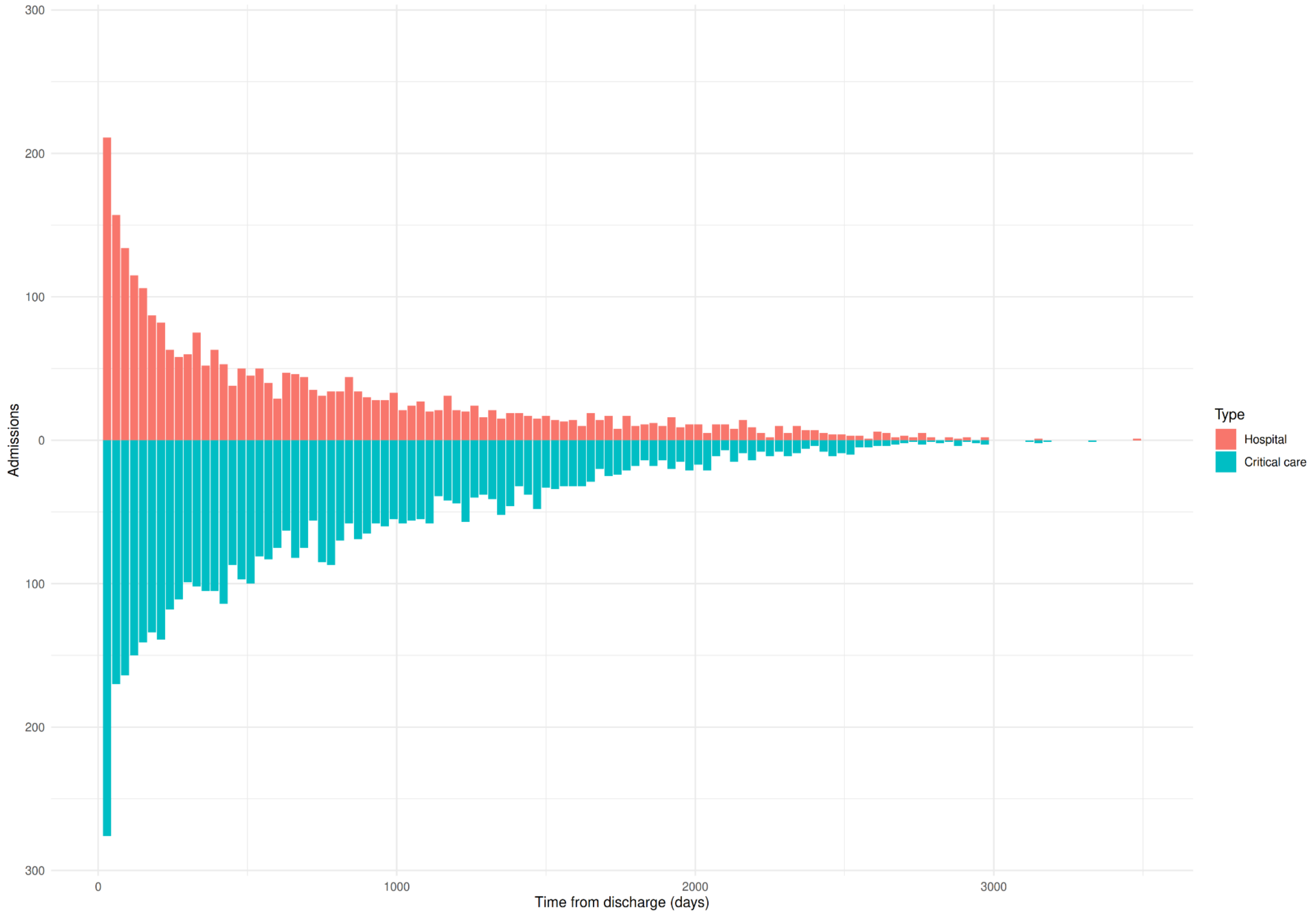

Online supplements are not copyedited prior to posting and the author(s) take full responsibility for the accuracy of all data.

e-Table 1: UK Biobank Identifiers used in this study

Outcome	Biobank Identifier
Overall Health Rating	2178
Frequency of depressed mood in the last two weeks	2050
Nervous feelings (Mental Health)	1970
Worrier/Anxious feelings	1980
Tense/highly strung	1990
Seen a GP for nerves, anxiety, tension or depression	2090
Sleeplessness/insomnia	1200
Miserableness	1930
Loneliness/isolation (Mental Health)	2020
Number in household	709
Leisure/social activities	6160
Frequency of friend/family visits	1031
Average household income before tax	738
Current employment status	6142
Own or rent accommodation lived in	680
Attendance/disability/mobility allowance	6416
Overall health rating	2178
Townsend deprivation index at recruitment	189
Ethnic Background	21000
Qualifications	6138
Consultant Speciality (Critical Care/ Intensive Therapy Unit)	41246/41245
Hand Grip Strength (left)	46
Hand Grip Strength (right)	47
Forced Vital Capacity	3062
MET minutes per week for all activity	22040

Online supplements are not copyedited prior to posting and the author(s) take full responsibility for the accuracy of all data.

e-Table 2: Comorbidities assessed

Hypothyroidism
Uncomplicated Diabetes
Myocardial Infarction
Neurological Disorders
Renal Disease
Deficiency Anaemia
Hypertension (with complications)
Chronic Pulmonary Disease
Rheumatic Disease
Valvular Disease
Alcohol Abuse
Peptic Ulcer Disease
Congestive Heart Failure
Cerebrovascular Disease
Obesity
Cardiac Arrhythmias
Peripheral Vascular Disorders
Mild Liver Disease
Pulmonary Circulation Disorders
Blood Loss Anaemia
Psychoses
Diabetes Complicated
Coagulopathy
Fluid and electrolyte disorders
Moderate or severe liver disease
Hemiplegia or Paraplegia
Drug abuse
Dementia
Weight Loss
Diabetes with chronic complication
Clinical Depression

Online supplements are not copyedited prior to posting and the author(s) take full responsibility for the accuracy of all data.

e-Table 3: UK Biobank Assessment Centre variables utilised

Figure: Calculation of Social Isolation

Reference Morneau-Vaillancourt, G. Coleman, JRI. Purves, KL. Et al (2020) The genetic and environmental hierarchical structure of anxiety and depression in the UK Biobank. Depression and Anxiety; 37:512-520.

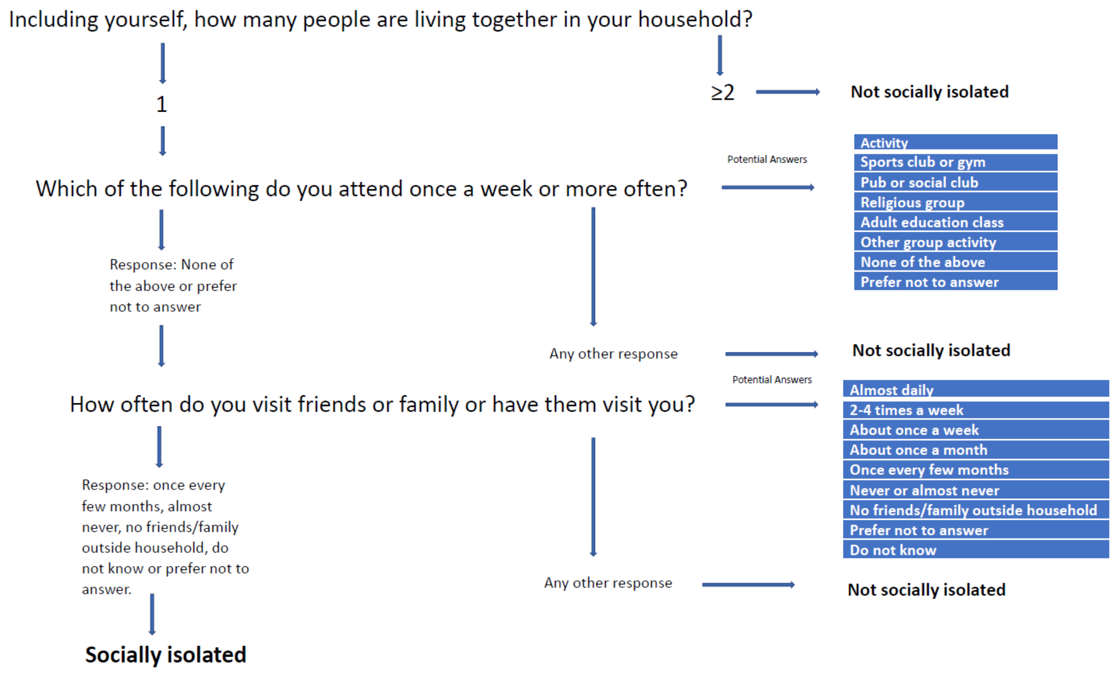

Tension

UK Biobank Question: Would you call yourself tense or highly strung?

Potential Answers:

1. Yes
2. No
3. Do not know
4. Prefer not to answer

Positive screen if answered yes

Nervous Feelings

UK Biobank Question: Would you call yourself a nervous person?

Potential Answers:

1. Yes
2. No
3. Do not know
4. Prefer not to answer

Positive screen if answered yes

Loneliness

UK Biobank Question: Do you often feel lonely?

Potential Answers:

5. Yes
6. No
7. Do not know
8. Prefer not to answer

Positive screen if answered yes

e-Table 4: Missing data Proportion of missing variables in UK Biobank

Variable	Missing data (%)
**Age**	0
**Gender**	0
**GP for anxiety/depression**	1.32
**Nervous**	2.8
**Tense feelings**	4.76
**Loneliness**	2.06
**Overall health**	1.64
**Employment status**	0.29
**Government allowances**	0.35
**Number in household**	1.16
**Hand grip strength**	0.96
**Forced Vital Capacity**	13.21
**Summed Minutes Activity**	23.59
**Length of stay (full)**	0
**Emergency admission**	0.03
**Townsend deprivation index**	0.19
**Ethnic background**	0.64
**Qualifications**	0.29
**Smoking status**	0.29

Online supplements are not copyedited prior to posting and the author(s) take full responsibility for the accuracy of all data.

e-Table 5: Adjusted Mortality Model (Removing all in-hospital variables)

Variable	Response	Unadjusted (95% CI)	Unadjusted p value	Adjusted (95% CI)	Adjusted p value
Age		1.06 (1.04-1.07)	<0.001	1.06 (1.04-1.07)	**<0.001**
Critical Care		1.31 (1.11- 1.56)	0.001	1.04 (0.86-1.26)	0.68
Gender	Male	1.31 (1.10- 1.56)	0.002	1.38 (1.11- 1.71)	**0.004**
GP for anxiety/depression		1.16 (0.98- 1.38)	0.09	0.91 (0.74- 1.11)	0.35
Nervous feelings		1.22 (1.01- 1.47)	0.04	1.20 (0.96- 1.50)	0.12
Tense feelings		1.23 (1.00- 1.51)	0.05	0.99 (0.78- 1.26)	0.96
Loneliness		1.02 (0.84- 1.23)	0.87		
**Overall health**	Fair	0.73 (0.57- 0.94)	0.01	0.87 (0.66- 1.15)	0.34
	Good	0.56 (0.44- 0.71)	< 0.001	0.77 (0.57- 1.04)	0.09
	Excellent	0.30 (0.20- 0.46)	< 0.001	0.49 (0.30- 0.78)	**0.003**
**Employment status**	Unable to work	1.49 (0.60- 3.69)	0.39		
	Vocational	1.35 (0.36- 5.04)	0.65		
	Purposeful	1.38 (0.57- 3.33)	0.47		
**Government allowances**	Blue badge	1.76 (1.04-3.00)	0.04	1.19 (0.69- 2.07)	0.53
	Allowances	1.73 (1.40-2.15)	< 0.001	0.97 (0.75-1.26)	0.82
Number in household		0.84 (0.78-0.91)	< 0.001	0.92 (0.85- 0.998)	**0.046**
Hand grip strength		0.99 (0.99- 1.00)	0.18		
Forced vital capacity		0.82 (0.75- 0.89)	< 0.001	0.86 (0.77- 0.96)	**0.007**
Summed minutes activity		1.0 (1- 1)	0.01	1 (1-1.00)	0.52
Townsend deprivation index		1.06 (1.03-1.08)	< 0.001	1.04 (1.01- 1.07)	**0.005**
**Ethnic background**	Mixed	0.40 (0.1- 1.59)	0.19		
	South Asian	1.12 (0.36- 3.50)	0.84		
	Black	1.13 (0.59-2.18)	0.72		
	Chinese	0.54 (0.08- 3.80)	0.53		
	Others	0.34 (0.05- 2.42)	0.28		
	Indian	1.24 (0.64- 2.39)	0.53		
	Pakistani	1.10 (0.35- 3.42)	0.87		
**Qualifications**	CSEs	0.47 (0.27- 0.81)	0.007	0.76 (0.44- 1.32)	0.33
	O levels/GCSEs	0.64 (0.48-0.86)	0.003	0.94 (0.70- 1.27)	0.69
	NVQ/ HND or HNC	0.61 (0.45-0.82)	0.001	0.84 (0.61- 1.14)	0.25
	A levels/AS levels	0.61 (0.42-0.9)	0.012	0.93 (0.63 -1.38)	0.73
	Other professional	0.57 (0.43-0.75)	< 0.001	0.80 (0.60- 1.07)	0.13
	College/University	0.72 (0.57-0.90)	0.004	1.11 (0.87-1.41)	0.40
**Smoking status**	Current	1.89 (1.52- 2.36)	< 0.001	1.81 (1.42- 2.31)	**< 0.001**
	Previous	1.41 (1.16- 1.71)	< 0.001	1.24 (1.01-1.51)	**0.04**

Online supplements are not copyedited prior to posting and the author(s) take full responsibility for the accuracy of all data.

**Comorbidities**				
Hypothyroidism	0.85 (0.60- 1.19)	0.34		
Cardiac arrhythmias	1.37 (1.05-1.79)	0.02	1.06 (0.8- 1.41)	0.69
Deficiency anaemia	1.52 (1.03- 2.26)	0.04	1 (0.66-1.52)	0.99
Chronic pulmonary disease	1.45 (1.18- 1.77)	< 0.001	0.98 (0.79- 1.23)	0.87
Diabetes (no complications)	1.36 (1.12-1.66)	0.002	1.02 (0.82- 1.27)	0.86
Myocardial infarction	1.17 (0.90- 1.51)	0.24		
Neurological disorders	2.45 (1.9- 3.15)	< 0.001	2.17 (1.63- 2.88)	**< 0.001**
Pulmonary circulation	1.66 (1.10- 2.5)	0.02	1.44 (0.94- 2.22)	0.1
Cerebrovascular disease	1.28 (1.0- 1.65)	0.05	0.95 (0.73- 1.24)	0.71
Obesity	1.05 (0.79- 1.4)	0.75		
Alcohol abuse	1.25 (0.95- 1.63)	0.11		
Congestive heart failure	1.83 (1.42- 2.37)	< 0.001	1.31 (0.98- 1.74)	0.06
Fluid/electrolyte disorders	1.66 (1.28- 2.14)	< 0.001	1.07 (0.81- 1.41)	0.64
Hypertension complicated	1.80 (1.21- 2.70)	0.004	0.74 (0.42- 1.30)	0.29
Renal disease	1.81 (1.35- 2.41)	< 0.001	1.49 (1.01- 2.22)	**0.05**
Valvular disease	1.32 (0.99- 1.77)	0.06	1.01 (0.74- 1.39)	0.93
Peripheral vascular disorder	1.37 (1.04- 1.81)	0.02	0.85 (0.63- 1.15)	0.29
Weight loss	2.27 (1.51- 3.42)	< 0.001	1.62 (1.6- 2.5)	**0.03**
Hemiplegia or paraplegia	1.0 (0.56- 1.77)	0.995		
Dementia	4.97 (3.32- 7.44)	< 0.001	3.07 (2.0- 4.72)	**< 0.001**
Peptic ulcer disease	1.55 (1.02- 2.35)	0.04	1.08 (0.69- 1.71)	0.73
Diabetes (complicated)	3.15 (2.15- 4.61)	< 0.001	1.89 (1.2- 2.99)	**0.006**
Rheumatic disease	1.35 (0.93- 1.97)	0.11		
Depression	1.7 (1.34- 2.16)	< 0.001	1.49 (1.14- 1.95)	**0.004**
Mild liver disease	2.45 (1.82- 3.29)	< 0.001	1.92 (1.35- 2.71)	**< 0.001**
Moderate/ severe liver disease	3.39 (2.03- 5.66)	< 0.001	1.82 (0.95- 3.49)	0.07
Coagulopathy	1.64 (0.94- 2.84)	0.08	1.08 (0.57-2.04)	0.81
Blood loss anaemia	0.55 (0.08- 3.93)	0.55		
Psychoses	1.41 (0.77- 2.55)	0.26		
Drug abuse	1.49 (0.48- 4.64)	0.49		

Online supplements are not copyedited prior to posting and the author(s) take full responsibility for the accuracy of all data.

e-Table 6: Adjusted Mortality Model (Removing variables with greater than 10% missing data)

Variable	Response	Unadjusted (95% CI)	Unadjusted p value	Adjusted (95% CI)	Adjusted p value
Age		1.06 (1.04-1.07)	<0.001	1.06 (1.05-1.08)	**<0.001**
Critical Care		1.31 (1.11- 1.56)	0.001	1.06 (0.88-1.29)	0.53
Gender	Male	1.31 (1.10- 1.56)	0.002	1.15 (0.96- 1.38)	0.14
GP for anxiety/depression		1.16 (0.98- 1.38)	0.09	0.91 (0.75- 1.12)	0.37
Nervous feelings		1.22 (1.01- 1.47)	0.04	1.19 (0.96- 1.49)	0.12
Tense feelings		1.23 (1.00- 1.51)	0.05	1.01 (0.79- 1.28)	0.94
Loneliness		1.02 (0.84- 1.23)	0.87		
**Overall health**	Fair	0.73 (0.57- 0.94)	0.01	0.86 (0.65- 1.13)	0.27
	Good	0.56 (0.44- 0.71)	< 0.001	0.74 (0.55- 0.99)	0.05
	Excellent	0.30 (0.20- 0.46)	< 0.001	0.45 (0.28- 0.72)	**<0.001**
**Employment status**	Unable to work	1.49 (0.60- 3.69)	0.39		
	Vocational	1.35 (0.36- 5.04)	0.65		
	Purposeful	1.38 (0.57- 3.33)	0.47		
**Government allowances**	Blue badge	1.76 (1.04-3.00)	0.04	1.27 (0.73- 2.20)	0.34
	Allowances	1.73 (1.40-2.15)	< 0.001	0.99 (0.76-1.28)	0.94
Number in household		0.84 (0.78-0.91)	< 0.001	0.92 (0.85- 1.00)	0.06
Hand grip strength		0.99 (0.99- 1.00)	0.18		
Emergency Admission		1.52 (1.25-1.86)	<0.001	1.35 (1.10-1.67)	**0.005**
Surgical Admission		1.12 (0.86-1.45)	0.42		
Townsend deprivation index		1.06 (1.03-1.08)	< 0.001	1.04 (1.01- 1.07)	**0.003**
Length of Stay (Full)		1 (1-1)	0.07	1 (1-1)	**0.003**
**Ethnic background**	Mixed	0.40 (0.1- 1.59)	0.19		
	South Asian	1.12 (0.36- 3.50)	0.84		
	Black	1.13 (0.59-2.18)	0.72		
	Chinese	0.54 (0.08- 3.80)	0.53		
	Others	0.34 (0.05- 2.42)	0.28		
	Indian	1.24 (0.64- 2.39)	0.53		
	Pakistani	1.10 (0.35- 3.42)	0.87		
**Qualifications**	CSEs	0.47 (0.27- 0.81)	0.007	0.74 (0.42- 1.29)	0.29
	O levels/GCSEs	0.64 (0.48-0.86)	0.003	0.91 (0.68- 1.23)	0.56
	NVQ/ HND or HNC	0.61 (0.45-0.82)	0.001	0.82 (0.60- 1.12)	0.21
	A levels/AS levels	0.61 (0.42-0.9)	0.012	0.93 (0.58 -1.02)	0.73
	Other professional	0.57 (0.43-0.75)	< 0.001	0.77 (0.58- 1.02)	0.07
	College/University	0.72 (0.57-0.90)	0.004	1.06 (0.83-1.34)	0.65

Online supplements are not copyedited prior to posting and the author(s) take full responsibility for the accuracy of all data.

**Smoking status**	Current	1.89 (1.52- 2.36)	< 0.001	1.84 (1.44- 2.35)	**< 0.001**
	Previous	1.41 (1.16- 1.71)	< 0.001	1.23 (1.00-1.50)	**0.049**
**Comorbidities**					
Hypothyroidism		0.85 (0.60- 1.19)	0.34		
Cardiac arrhythmias		1.37 (1.05-1.79)	0.02	1.07 (0.81- 1.42)	0.64
Anaemia Deficiency		1.52 (1.03- 2.26)	0.04	0.96 (0.63-1.46)	0.85
Chronic pulmonary disease		1.45 (1.18- 1.77)	< 0.001	1.03 (0.83- 1.28)	0.79
Diabetes (no complications)		1.36 (1.12-1.66)	0.002	1.08 (0.87- 1.35)	0.49
Myocardial infarction		1.17 (0.90- 1.51)	0.24		
Neurological disorders		2.45 (1.9- 3.15)	< 0.001	2.11 (1.59- 2.80)	**< 0.001**
Pulmonary circulation		1.66 (1.10- 2.5)	0.02	1.45 (0.94- 2.22)	0.09
Cerebrovascular disease		1.28 (1.0- 1.65)	0.05	0.93 (0.71- 1.21)	0.59
Obesity		1.05 (0.79- 1.4)	0.75		
Alcohol abuse		1.25 (0.95- 1.63)	0.11		
Congestive heart failure		1.83 (1.42- 2.37)	< 0.001	1.28 (0.96- 1.70)	0.09
Fluid/electrolyte disorders		1.66 (1.28- 2.14)	< 0.001	1.02 (0.77- 1.35)	0.90
Hypertension complicated		1.80 (1.21- 2.70)	0.004	0.72 (0.41- 1.26)	0.24
Renal disease		1.81 (1.35- 2.41)	< 0.001	1.61 (1.08- 2.38)	**0.02**
Valvular disease		1.32 (0.99- 1.77)	0.06	1.03 (0.75- 1.42)	0.84
Peripheral vascular disorder		1.37 (1.04- 1.81)	0.02	0.89 (0.66- 1.20)	0.44
Weight loss		2.27 (1.51- 3.42)	< 0.001	1.58 (1.03- 2.43)	**0.04**
Hemiplegia or paraplegia		1.0 (0.56- 1.77)	0.995		
Dementia		4.97 (3.32- 7.44)	< 0.001	2.94 (1.92- 4.50)	**< 0.001**
Peptic ulcer disease		1.55 (1.02- 2.35)	0.04	1.10 (0.69- 1.74)	0.69
Diabetes (complicated)		3.15 (2.15- 4.61)	< 0.001	1.81 (1.14- 2.87)	**0.01**
Rheumatic disease		1.35 (0.93- 1.97)	0.11		
Depression		1.7 (1.34- 2.16)	< 0.001	1.47 (1.13- 1.93)	**0.01**
Mild liver disease		2.45 (1.82- 3.29)	< 0.001	1.98 (1.40- 2.79)	**< 0.001**
Moderate/ severe liver disease		3.39 (2.03- 5.66)	< 0.001	1.69 (0.88- 3.23)	0.11
Coagulopathy		1.64 (0.94- 2.84)	0.08	1.02 (0.55-1.91)	0.95
Blood loss anaemia		0.55 (0.08- 3.93)	0.55		
Psychoses		1.41 (0.77- 2.55)	0.26		
Drug abuse		1.49 (0.48- 4.64)	0.49		

Online supplements are not copyedited prior to posting and the author(s) take full responsibility for the accuracy of all data.

e-Table 7: Causes of death in the hospital and critical care cohorts

	Hospital Cohort (n=261)	Critical care Cohort (n=285)
Cardiac or vascular	36	54
Neuro/stroke (non-cancer)	57	25
Respiratory (non-cancer)	19	33
GI (cancer)	26	23
Cancer (other)	22	26
Hepatic (non-cancer)	10	11
Respiratory (cancer)	10	10
Neuro/stroke (cancer)	8	9
COVID-19	7	*
Diabetes mellitus	*	6
Other	66	87

*Denotes a value less than five

Online supplements are not copyedited prior to posting and the author(s) take full responsibility for the accuracy of all data.

e-Table 8: Adjusted and unadjusted analyses alongside incident rate ratios for hospital readmission in the year following ICU

Variable	Response	Unadjusted (95% CI)	Unadjusted p value	Adjusted (95% CI)	Adjusted p value
Age		1.01 (0.99-1.01)	0.24		
Critical Care		1.71 (1.51- 1.95)	<0.001	1.27 (1.12-1.45)	**<0.001**
Gender	Male	1.03 (0.90- 1.17)	0.67		
GP for anxiety/depression		1.03 (0.90- 1.17)	0.71		
Nervous feelings		1.06 (0.91- 1.23)	0.46		
Tense feelings		1.27 (1.08- 1.49)	0.004	0.96 (0.81- 1.12)	0.58
Loneliness		1.01 (0.87- 1.18)	0.87		
**Overall health**	Fair	0.70 (0.56- 0.87)	0.001	0.83 (0.66- 1.04)	0.1
	Good	0.59 (0.48- 0.72)	< 0.001	0.75 (0.59- 0.94)	**0.01**
	Excellent	0.48 (0.36- 0.64)	< 0.001	0.76 (0.56- 1.02)	0.07
**Employment status**	Unable to work	0.76 (0.42- 1.39)	0.37		
	Vocational	0.92 (0.36- 2.31)	0.85		
	Purposeful	0.80 (0.45- 1.43)	0.45		
**Government allowances**	Blue badge	1.28 (0.79-2.06)	0.31	1.14 (0.74- 1.78)	0.55
	Allowances	1.35 (1.11-1.63)	0.002	1.03 (0.83-1.27)	0.81
Number in household		0.96 (0.92-1.00)	< 0.06	0.97 (0.93- 1.01)	0.15
Hand grip strength		1.01 (1- 1.01)	0.04	1 (0.99-1.01)	0.88
Forced vital capacity		0.97 (0.91- 1.04)	0.36		
Summed minutes activity		1.0 (1- 1)	0.68		
Length of stay (full)		1 (1- 1)	0.50		
Emergency admission		1.01 (0.87- 1.17)	0.91		
Surgical admission		1.30 (1.06-1.59)	0.01	1.19 (0.98-1.44)	0.07
Townsend deprivation index		1.01 (0.99-1.03)	0.27		
**Ethnic background**	Mixed	0.73 (0.35- 1.54)	0.41	0.91 (0.45-1.83)	0.79
	South Asian	0.60 (0.22- 1.67)	0.33	0.69 (0.26-1.81)	0.45
	Black	0.71 (0.41-1.24)	0.23	0.70 (0.41-1.18)	0.18
	Chinese	13.13 (4.59- 37.6)	<0.001	12.27 (4.53-33.2)	**<0.001**
	Others	0.44 (0.17- 1.12)	0.9	0.55 (0.22-1.35)	0.19
	Indian	1.35 (0.8- 2.29)	0.27	1.08 (0.63-1.83)	0.79
	Pakistani	0.61 (0.23- 1.63)	0.33	0.73 (0.52-1.19)	0.51
**Qualifications**	CSEs	0.66 (0.46- 0.95)	0.02	0.73 (0.52- 1.02)	0.07
	O levels/GCSEs	1.02 (0.82-1.27)	0.86	0.97 (0.78- 1.19)	0.74
	NVQ/ HND or HNC	0.82 (0.66-1.04)	0.1	0.96 (0.77- 1.19)	0.71
	A levels/AS levels	1.43 (1.09-1.89)	0.01	0.94 (0.72 1.24)	0.67
	Other professional	0.86(0.70-1.06)	0.16	0.97 (0.80- 1.18)	0.75
	College/University	0.89(0.74-1.07)	0.21	0.88 (0.73-1.05)	0.15

Online supplements are not copyedited prior to posting and the author(s) take full responsibility for the accuracy of all data.

**Smoking status**	Current	1.29 (1.08- 1.54)	0.005	1.30 (1.09- 1.55)	**0.004**
	Previous	1.04 (0.90- 1.20)	0.62	1.15 (1.00-1.32)	**0.05**
**Comorbidities**					
Hypothyroidism		1.01 (0.80- 1.28)	0.92		
Cardiac arrhythmias		1.02 (0.82-1.27)	0.85		
Deficiency anaemia		1.31 (0.95- 1.82)	0.10	1.17 (0.86-1.60)	0.31
Chronic pulmonary disease		0.87 (0.73- 1.03)	0.12		
Diabetes (no complications)		1.14 (0.97-1.35)	0.11		
Myocardial infarction		0.75 (0.61- 0.92)	0.006	0.75 (0.61-0.92)	**0.007**
Neurological disorders		0.87(0.69- 1.13)	0.34		
Pulmonary circulation		0.85 (0.59-1.21)	0.38		
Cerebrovascular disease		0.62 (0.50-0.77)	<0.001	0.66 (0.54- 0.81)	**<0.001**
Obesity		1.31 (1.05- 1.62)	0.02	0.93 (0.75-1.15)	0.51
Alcohol abuse		0.78 (0.62-0.97)	0.03	0.67 (0.54-0.84)	<**0.001**
Congestive heart failure		1.34 (1.07- 1.68)	0.01	0.98 (0.77- 1.24)	0.87
Fluid/electrolyte disorders		1.97 (1.61- 2.42)	< 0.001	1.33 (1.08- 1.64)	**0.008**
Hypertension complicated		5.71 (4.07- 8.01)	<0.001	1.84(1.16- 2.91)	**0.009**
Renal disease		4.15 (3.30- 5.23)	< 0.001	1.48(1.08- 2.03)	**0.02**
Valvular disease		1.48 (1.17- 1.87)	0.001	1.18 (0.93- 1.49)	0.18
Peripheral vascular disorder		1.83 (1.43- 2.28)	<0.001	1.01 (0.80- 1.28)	0.91
Weight loss		1.51 (1.03- 2.02)	0.04	1.59 (1.11- 2.28)	**0.01**
Hemiplegia or paraplegia		0.86 (0.55- 1.35)	0.51		
Dementia		0.62 (0.36- 1.04)	0.07	0.64 (0.39- 1.06)	**0.08**
Peptic ulcer disease		1.26 (0.88- 1.79)	0.20		
Diabetes (complicated)		2.55 (1.7- 3.82)	< 0.001	1.08 (0.72- 1.64)	0.70
Rheumatic disease		1.03 (0.75- 1.41)	0.85		
Depression		1.29 (1.04- 1.60)	0.02	1.11 (0.90- 1.38)	0.33
Mild liver disease		3.11 (2.37- 4.06)	< 0.001	2.16 (1.65- 2.82)	**< 0.001**
Moderate/ severe liver disease		1.19 (0.72- 1.98)	< 0.50		
Coagulopathy		0.8 (0.50- 1.29)	0.36		
Blood loss anaemia		1.34 (0.44- 4.06)	0.61		
Psychoses		0.71 (0.41- 1.24)	0.23		
Drug abuse		9.71 (4.02- 23.47)	<0.001	2.20 (0.90-5.34)	0.08

Online supplements are not copyedited prior to posting and the author(s) take full responsibility for the accuracy of all data.

e-Table 9: Adjusted and unadjusted analyses alongside Odd Ratio of hospital readmission risk in the year following ICU discharge

Variable	Response	Unadjusted (95% CI)	Unadjusted p value	Adjusted (95% CI)	Adjusted p value
Age		1.0 (0.99-1.01)	0.8		
Critical Care		144 (1.25- 1.66)	<0.001	1.29 (1.11-1.50)	**<0.001**
Gender	Male	1.14 (0.99- 1.31)	0.07	1.13 (0.97-1.32)	0.11
GP for anxiety/depression		1.2 (1.04- 1.39)	0.02	1.06 (0.9-1.25)	0.46
Nervous feelings		1.02 (0.87- 1.21)	0.78		
Tense feelings		1.06 (0.77- 1.27)	0.55		
Loneliness		1.14 (0.97- 1.35)	0.12		
**Overall health**	Fair	0.66 (0.52- 0.85)	0.001	0.79 (0.60- 1.04)	0.09
	Good	0.54 (0.42- 0.68)	< 0.001	0.75 (0.57- 0.99)	**0.05**
	Excellent	0.54 (0.39- 0.73)	< 0.001	0.80 (0.56- 1.13)	0.21
**Employment status**	Unable to work	0.85 (0.43- 1.65)	0.62		
	Vocational	0.81 (0.29- 2.26)	0.69		
	Purposeful	0.67 (0.35- 1.28)	0.23		
**Government allowances**	Blue badge	1.64 (0.96-2.80)	0.07	1.26 (0.73- 2.19)	0.41
	Allowances	1.47 (1.18-1.82)	<0.001	1.07 (0.83-1.37)	0.62
Number in household		0.98 (0.94-1.03)	< 0.49		
Hand grip strength		1.0 (0.99-1.01)	0.62		
Forced vital capacity		0.98 (0.91- 1.05)	0.56		
Summed minutes activity		1.0 (1- 1)	0.44		
Length of stay (full)		1 (1- 1)	0.51		
Emergency admission		0.97 (0.82- 1.13)	0.68		
Surgical admission		1.14 (0.92-1.42)	0.24		
Townsend deprivation index		1.03 (1.01-1.05)	0.007	1.01(0.99-1.04)	0.28
**Ethnic background**	Mixed	2.08 (0.92- 4.71)	0.08	2.25 (0.97-5.2)	0.06
	South Asian	1.56 (0.54- 4.5)	0.41	1.4 (0.47-4.15)	0.54
	Black	1.22 (0.68-2.2)	0.51	1.08 (0.59-1.99)	0.80
	Chinese	0.33 (0.07- 1.61)	0.17	0.48 (0.10-2.32)	0.36
	Others	0.68 (0.27- 1.74)	0.42	0.57 (0.21-1.51)	0.26
	Indian	1.12 (0.61- 2.04)	0.72	0.95 (0.50-1.78)	0.86
	Pakistani	0.78(0.28- 2.19)	0.64	0.72 (0.25-2.07)	0.54
**Qualifications**	CSEs	0.69 (0.47- 1.01)	0.06	0.71 (0.47- 1.05)	0.09
	O levels/GCSEs	0.71 (0.56-0.92)	0.08	0.74 (0.58- 0.96)	0.02
	NVQ/ HND or HNC	1.06 (0.83-1.36)	0.64	1.09 (0.85- 1.41)	0.49
	A levels/AS levels	0.78 (0.57-1.06)	0.11	0.80 (0.58 1.12)	0.19
	Other professional	0.99 (0.79-1.25)	0.96	1.09 (0.86- 1.37)	0.49
	College/University	0.85 (0.69-1.03)	0.10	0.91 (0.74-1.13)	0.39

Online supplements are not copyedited prior to posting and the author(s) take full responsibility for the accuracy of all data.

**Smoking status**	Current	1.29 (1.07- 1.58)	0.009	1.19 (0.96- 1.47)	0.11
	Previous	1.17 (1.00- 1.37)	0.05	1.12 (0.95-1.32)	0.18
**Comorbidities**					
Hypothyroidism		1.27 (0.98- 1.65)	0.07	1.22 (0.93-1.60)	0.15
Cardiac arrhythmias		1.21 (0.88-1.42)	0.35		
Deficiency anaemia		1.93 (1.33- 2.79)	<0.001	1.52 (1.03-2.25)	**0.03**
Chronic pulmonary disease		1.1 (0.92- 1.33)	0.30		
Diabetes (no complications)		1.10 (0.91-1.31)	0.30		
Myocardial infarction		0.99 (0.80- 1.24)	0.94		
Neurological disorders		1.09 (0.84- 1.43)	0.52		
Pulmonary circulation		1.02 (0.69-1.5)	0.92		
Cerebrovascular disease		0.80 (0.64-1.0)	0.05	0.78 (0.62- 0.98)	0.03
Obesity		1.29 (1.02-1.64)	0.04	1.09 (0.84-1.40)	0.52
Alcohol abuse		1.08 (0.85-1.37)	0.53		
Congestive heart failure		1.21 (0.94- 1.55)	0.14		
Fluid/electrolyte disorders		1.04 (0.82- 1.31)	0.76		
Hypertension complicated		1.44 (0.94-2.22)	0.09	0.99 (0.62-1.58)	0.98
Renal disease		1.23 (0.92- 1.63)	0.17		
Valvular disease		1.29 (0.99- 1.68)	0.06	1.18 (0.90- 1.55)	0.24
Peripheral vascular disorder		1.27 (1.98- 1.64)	0.07	1.01 (0.77- 1.32)	0.96
Weight loss		1.64 (1.06- 2.52)	0.03	1.33 (0.85- 2.08)	0.22
Hemiplegia or paraplegia		1.31 (0.81- 2.13)	0.27		
Dementia		0.68 (0.39-1.18)	0.17		
Peptic ulcer disease		1.52 (1.02- 2.25)	0.04	1.08 (0.72-1.64)	0.70
Diabetes (complicated)		1.56 (0.97- 2.52)	0.07	1.04 (0.62- 1.76)	0.87
Rheumatic disease		1.39 (0.98- 1.96)	0.06	1.30 (0.90-1.86)	0.16
Depression		1.52 (1.20- 1.93)	<0.001	1.28 (0.98- 1.66)	0.07
Mild liver disease		1.72 (1.24- 2.38)	0.001	1.49 (1.06- 2.10)	**0.02**
Moderate/ severe liver disease		1.16 (0.67- 2.04)	0.59		
Coagulopathy		1.05 (0.63- 1.75)	0.84		
Blood loss anaemia		4.67 (0.99- 22.00)	0.05		
Psychoses		0.74 (0.41- 1.35)	0.33		
Drug abuse		0.73 (0.24- 2.22)	0.57		

Online supplements are not copyedited prior to posting and the author(s) take full responsibility for the accuracy of all data.
